# Laropiprant attenuates EP_3_ and TP prostanoid receptor-mediated thrombus formation

**DOI:** 10.1186/2050-6511-13-S1-A14

**Published:** 2012-09-17

**Authors:** Sonia Philipose, Viktória Kónya, Mirjana Lazarević, Lisa M Pasterk, Gunther Marsche, Sasa Frank, Bernhard A Peskar, Ákos Heinemann, Rufina Schuligoi

**Affiliations:** 1 Institute of Experimental and Clinical Pharmacology, Medical University of Graz, 8010 Graz Austria; 2 Institute of Molecular Biology and Biochemistry, Medical University of Graz, 8010 Graz, Austria

## Background

The use of the lipid-lowering agent niacin is hampered by a frequent flush response which is largely mediated by prostaglandin (PG) D_2_. Therefore, concomitant administration of the D-type prostanoid (DP) receptor antagonist laropiprant has been proposed to be a useful approach in preventing niacin-induced flush. However, antagonizing PGD_2_, which is a potent inhibitor of platelet aggregation, might pose the risk of atherothrombotic events in cardiovascular disease. Therefore, we investigated the effects of laropiorant on platelet function.

## Methods

Platelet aggregation assays were performed *ex vivo* using a platelet aggregation analyser (Aggregometer II). Blood from healthy human donors was used to obtain platelet-rich plasma. The expression of P-selectin and activation of glycoprotein IIb/IIIa was examined using CD62P and PAC1 antibodies, respectively, by direct flow cytometry. *In vitro* thrombus formation was assessed by flowing whole blood on collagen-coated Cellix biochips at −30 dyn/cm^2^ using the Mirus nanopump.

## Results

*In vitro* treatment of platelets with laropiprant prevented the inhibitory effects of PGD_2_ on platelet function, *i.e.* platelet aggregation, P-selectin expression, activation of glycoprotein IIb/IIIa and thrombus formation. In contrast, laropiprant did not prevent the inhibitory effects of acetylsalicylic acid or niacin on thrombus formation. At higher concentrations, laropiprant by itself attenuated platelet activation induced by thromboxane (TP) and E-type prostanoid (EP)-3 receptor stimulation, as demonstrated in assays of platelet aggregation, P-selectin expression, and activation of glycoprotein IIb/IIIa. Inhibition of platelet function exerted by EP_4_ or I-type prostanoid (IP) receptors was not affected by laropiprant.

## Conclusions

These *in vitro* data suggest that niacin/laropiprant for the treatment of dyslipidemias might have a beneficial profile with respect to platelet function and thrombotic events in vascular disease.

